# Cloning and Functional Analysis of MADS-box Genes, *TaAG-A* and *TaAG-B*, from a Wheat K-type Cytoplasmic Male Sterile Line

**DOI:** 10.3389/fpls.2017.01081

**Published:** 2017-06-20

**Authors:** Wenlong Yang, Xueyuan Lou, Juan Li, Mingyu Pu, Ameer A. Mirbahar, Dongcheng Liu, Jiazhu Sun, Kehui Zhan, Lixiong He, Aimin Zhang

**Affiliations:** ^1^The State Key Laboratory of Plant Cell and Chromosome Engineering, Institute of Genetics and Developmental Biology, Chinese Academy of SciencesBeijing, China; ^2^College of Forestry, Henan Agricultural UniversityZhengzhou, China; ^3^College of Life Sciences, Hunan Agricultural UniversityChangsha, China; ^4^Department of Botany, Shah Abdul Latif UniversityKhairpur, Pakistan; ^5^The Collaborative Center for Grain Crops in Henan, College of Agronomy, Henan Agricultural UniversityZhengzhou, China

**Keywords:** wheat, male sterile, MADS-box gene, cloning, functional analysis

## Abstract

Wheat (*Triticum aestivum* L.) is a major crop worldwide. The utilization of heterosis is a promising approach to improve the yield and quality of wheat. Although there have been many studies on wheat cytoplasmic male sterility, its mechanism remains unclear. In this study, we identified two MADS-box genes from a wheat K-type cytoplasmic male sterile (CMS) line using homology-based cloning. These genes were localized on wheat chromosomes 3A and 3B and named *TaAG-A* and *TaAG-B*, respectively. Analysis of *TaAG-A* and *TaAG-B* expression patterns in leaves, spikes, roots, and stems of Chinese Spring wheat determined using quantitative RT-PCR revealed different expression levels in different tissues. *TaAG-A* had relatively high expression levels in leaves and spikes, but low levels in roots, while *TaAG-B* had relatively high expression levels in spikes and lower expression in roots, stems, and leaves. Both genes showed downregulation during the mononucleate to trinucleate stages of pollen development in the maintainer line. In contrast, upregulation of *TaAG-B* was observed in the CMS line. The transcript levels of the two genes were clearly higher in the CMS line compared to the maintainer line at the trinucleate stage. Overexpression of *TaAG-A* and *TaAG-B* in *Arabidopsis* resulted in phenotypes with earlier reproductive development, premature mortality, and abnormal buds, stamens, and stigmas. Overexpression of *TaAG-A* and *TaAG-B* gives rise to mutants with many deformities. Silencing *TaAG-A* and *TaAG-B* in a fertile wheat line using the virus-induced gene silencing (VIGS) method resulted in plants with green and yellow striped leaves, emaciated spikes, and decreased selfing seed set rates. These results demonstrate that *TaAG-A* and *TaAG-B* may play a role in male sterility in the wheat CMS line.

## Introduction

Wheat (*Triticum aestivum* L.) is a widely cultivated and consumed food crop worldwide. High yield and quality are the ultimate objectives of grain production. The utilization of heterosis is a promising approach to improving the yield and quality of wheat. Cytoplasmic male sterility, characterized by the inability to produce viable pollen while vegetative and female development remain unaffected (Duvick, 1959), is an essential tool in the utilization of heterosis. K-type cytoplasmic male sterile (CMS) lines (male sterile lines with *Aegilops kotschyi* cytoplasm) are potential candidates for use in experiments because they suffer few side effects and have abundant restoring resources among common wheat cultivars (Tsunewaki et al., 1978). The mechanism of wheat male sterility remains unclear. The CMS phenotype is mainly reflected in flowers. The ABC(DE) model of flower development in higher plants proposes that class A, B, C, D, and E homeotic genes specify floral organ identity (Theißen, 2001). According to this model, class A and E genes in combination control the development of sepals; class A, B, and E genes control petal development; class B, C, and E genes control stamen development; and class C and E genes control carpel development. The wheat inflorescence (spike or ear) is composed of spikelets, whereas an individual wheat flower contains one pistil, three stamens, and two lodicules, which are modified petals. Though the floral organs of wheat differ from those of dicotyledons, the ABC(DE) model can also be applied to wheat flower development (Murai, 2013).

In the typical ABC model (Weigel and Meyerowitz, 1994) of *Arabidopsis*, *APETALA1* (*AP1*, Mandel et al., 1992) and *APETALA2* (*AP2*, Jofuku et al., 1994) are classified as A genes, *APETALA3* (*AP3*, Jack et al., 1992) and *PISTILLATA* (*PI*, Goto and Meyerowitz, 1994) are B genes, and *AGAMOUS* (*AG*, Yanofsky et al., 1990) is the only class C gene. With the exception of *AP2*, the other four genes *AP1*, *AP3*, *PI*, and *AG*, are all MADS-box genes. MADS-box genes encode a eukaryotic transcription factor family that plays a fundamental role in very diverse and important biological functions, including vegetative and reproductive development, floral organ development, and signal transduction (Ng and Yanofsky, 2001; De Bodt et al., 2003; Duan et al., 2006). In the past few decades, various proteins have been identified with highly conserved DNA binding and dimerized MADS-box domains (Schwarz-Sommer et al., 1990; Coen and Meyerowitz, 1991) in yeasts, plants, insects, nematodes, lower vertebrates, and mammals. Moreover, several ABC(DE) MADS-box genes, such as *WAG* (Meguro et al., 2003; Hirabayashi and Murai, 2009; Wei et al., 2011; Wang et al., 2015), *WAP1* (Murai et al., 2003), and *TaMADS1* (Zhao et al., 2005), have been identified and characterized in wheat. The MADS-box gene family was further split into type I (SRF-like) and type II (MEF2-like) genes through phylogenetic analysis. Type II proteins, which are also called MIKC-type proteins, are mostly found in higher plants (Münster et al., 1997). Some of them are related to male sterility (Ai et al., 2016).

MIKC-type proteins play a vital role in floral organ formation (Ng and Yanofsky, 2001). Our previous microarray results showed higher expression levels of a MIKC-type MADS-box transcription factor, Ta.4147, in a wheat K-type CMS line, in contrast to the maintainer line. To understand the relationship between the MADS-box gene and male sterility in the CMS line, in this study we identified two MADS-box genes from the uninucleate pollen of the CMS line using homology-based cloning. The two genes were named *TaAG-A* and *TaAG-B* based on their localization on wheat chromosomes 3A and 3B, respectively. The objective of the present study was to investigate the functions of the *TaAG-A* and *TaAG-B* genes. We conducted expression analysis of the genes in different tissues at different stages. Overexpression in *Arabidopsis* and silencing in a wheat fertile line using BSMV-VIGS were also performed. The results suggest that *TaAG-A* and *TaAG-B* may play central roles in male sterility of the wheat CMS line.

## Materials and Methods

### Plant Materials

A wheat K-type CMS line (Yumai 3, KA), a maintainer line (Yumai 3, KB), a restorer (Yumai 2) line, and F_1_ lines were grown in the experimental field of the Institute of Genetics and Developmental Biology, Chinese Academy of Sciences (Beijing, China) until the heading stage. Anthers at the mononucleate, dinucleate, and trinucleate stages were sampled, quickly frozen in liquid nitrogen, and stored at -80°C until RNA isolation.

Wheat variety Chinese Spring and 21 Chinese Spring nullisomic-tetrasomic lines were grown in a greenhouse at 22°C under a 16-h light/8-h dark regime. Young leaves were collected for DNA extraction. Roots, stems, flag leaves, and young spikes at the heading stage were sampled for RNA isolation.

*Arabidopsis* wild-type Columbia (Col-0) and transgenic pEarleyGate 101 plants (pEarleyGate 101-*TaAG-A* and pEarleyGate 101-*TaAG-B*) were grown in a greenhouse at 22°C under a 16-h light/8-h dark regime. Leaves were collected for RNA isolation.

Normal wheat and wheat inoculated with RNA_γb:TaAG-A_ or RNA_γb:TaAG-B_ were grown in a greenhouse at 25/15°C under a 16-h light/8-h dark regime. Spikes were collected for RNA isolation.

### Genomic DNA and RNA Extraction

Genomic DNA was extracted using the cetyltrimethylammonium bromide (CTAB) method (Saghai-Maroof et al., 1984). Total RNA was isolated using TRIzol reagent (Invitrogen, Carlsbad, CA, United States) and treated with RNase-free DNaseI (Promega, Madison, WI, United States) as described by Huang et al. (2012). First strand cDNA was synthesized using the FastQuant RT Kit (with gDNase) (TIANGEN, Beijing) following the manufacturer’s instructions.

### Cloning of Wheat MADS-box Genes

Ta.4147 was used to conduct a BLAST search of the *Triticum urartu* genome database^1^. Then two pairs of gene specific primers, TaMADS1.CF/CR and TaMADS2.CF/CR (Supplementary Table S1), were designed and used to clone MADS-box genes from the cDNA of anthers from the male sterile line at the mononucleate stage. The following PCR reaction conditions were used: 5 min at 94°C; followed by 35 cycles of 30 s at 94°C, 30 s at 58°C, and 1 min at 72°C; with a final extension at 72°C for 10 min using LA-*Taq* DNA polymerase (Takara, Biotechnology Co., Ltd., Dalian, China). The PCR products were individually cloned into pGEM-T easy vectors (Promega, Madison, WI, United States) and introduced into *Escherichia coli*. Ten positive independent clones were commercially sequenced. The GenBank/EMBL accession numbers are as follows: KX354939–KX354940. The sequences were analyzed using DNAMAN software and the Conserved Domain Search in NCBI^2^. The phylogenetic trees were drawn using Clustal W and MEGA software.

### Chromosomal Localization Analysis

Nulli-tetrasomic lines of Chinese Spring were employed to determine the chromosomal locations of the *TaMADS1* and *TaMADS2* genes with the gene-specific primers TaMADS1.LEF/LER and TaMADS2.LEF/LER (Supplementary Table S1). The PCR products were resolved by electrophoresis on a 1% agarose gel containing ethidium bromide and visualized under a UV spectrometer.

### Quantitative Real-time PCR Analysis

Quantitative real-time PCR (qRT-PCR) was performed to study relative gene expression levels using the LightCycler 480 system (Roche, Indianapolis, IN, United States) with SYBR Green I Master (Roche) according to the manufacturer’s protocol. Two pairs of gene-specific primers, TaMADS1.LEF/LER and TaMADS2.LEF/LER (Supplementary Table S1), were used for gene expression analysis with the *Ta4045* gene, which encodes ubiquinol-cytochrome C reductase iron-sulfur subunit as described by Paolacci et al. (2009), as a control. The expression profiles of *TaAG-A* and *TaAG-B* in various wheat organs (roots, stems, flag leaves, and young spikes) and at different stages (mononucleate, dinucleate, and trinucleate) were investigated. The qRT-PCR experiment was performed independently with three biological replicates and three technical replicates according to the method of Li et al. (2013).

### Transformation Assays

The entire coding sequences of the *TaAG-A* and *TaAG-B* genes were individually cloned into the pEarleyGate101 vector using the Gateway-compatible vector cloning system (Invitrogen, Carlsbad, CA, United States) to produce the plasmid constructs pEarleyGate 101- *TaAG-A* and pEarleyGate 101- *TaAG-B*. The constructs were used to transform *Arabidopsis* wild-type Col-0 by the *Agrobacterium*- mediated floral-dip method (Clough and Bent, 1998). Transgenic lines were selected on MS (Murashige and Skoog, 1962) plates with phosphinothricin (5 μg/L), and more than 20 transgenic lines were obtained. Transgenic plants were selected for phenotypic analysis and semi-quantitative RT-PCR with *Actin* as a control. The primers used for plasmid construction (TaAG-AB.101F/101R), transformant identification (101.F/R), and semi-quantitative RT-PCR (TaAG-AB.SF/SR, Actin.F/R) are listed in Supplementary Table S1.

### Virus-Induced Gene Silencing with the Barley Stripe Mosaic Virus

The *Nhe*I restriction site was introduced into *TaAG-A* and *TaAG-B* using the primers TaAG-A-*Nhe*I-F/R and TaAG-B-*Nhe*I-F/R (Supplementary Table S1). The amplified fragments were used to replace the GFP coding sequence in RNA_γb:GFP_, resulting in RNA_γb:TaAG-A_ and RNA_γb:TaAG-B_. The preparation of BSMV transcripts for wheat inoculation was consistent with the method of Zhou et al. (2007). The fourth leaf and spikes of inoculated plants were observed at 12, 16, and 20 days after inoculation. The relative transcript levels of the *TaAG-A* and *TaAG-B* genes in the spikes were assessed by semi-quantitative RT-PCR, which were performed independently with three biological replicates and three technical replicates.

## Results

### Cloning and Sequence Analysis of Two MADS-box Genes from a Wheat K-type CMS Line

**FIGURE 1:**
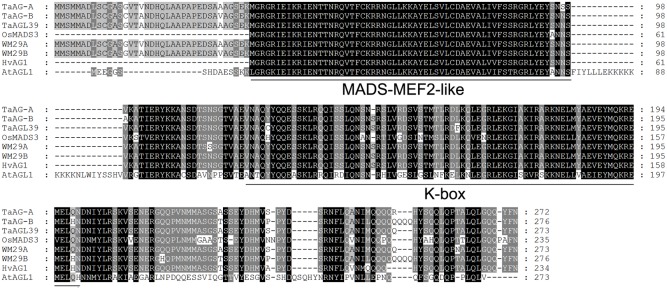
Multiple alignment of the TaMADS1 and TaMADS2 amino acid sequences. Wheat TaWM29A (CAM59076) and TaAGL39 (ABF57939), barley HvWM29B (ACB45306), *Brachypodium distachyon* BdMADS (XP_003565181), and rice OsMADS3 (ACY26070). Underlines indicate the conserved MADS-MEF2-like region and the K-box motif.

Our previous microarray results showed that the expression level of the MIKC-type MADS-box transcription factor Ta.4147 in the CMS line was higher than in the maintainer line. Therefore, the Ta.4147 sequence was used to conduct a BLAST search of the *T*. *urartu* genome database and we obtained a very similar sequence, TRIUR3_34584 (GenBank accession number KD145636). We designed gene-specific primers (Supplementary Table S1) and performed RT-PCR analysis with the purified RNA extracted from uninucleate pollen of the wheat K-type CMS line. The PCR products were separated on a 1% agarose gel and purified with the Agarose Gel DNA Purification kit (TIAGEN, Beijing) according to the manufacturer’s instructions. The purified fragment was then cloned into the pGEM T easy vector (Promega) and transformed into *E. coli* TOP10. Positive clones were selected for sequencing. The results of sequence analysis (Supplementary Table S2) revealed two types of sequences derived from positive clones. One sequence had an open reading frame (ORF) of 819 bp encoding a peptide of 272 amino acid residues with a molecular weight of 30.8 kDa and a pI of 9.072. The other type had an ORF of 831 bp encoding a peptide of 276 amino acid residues with a molecular weight of 31.4 kDa and a pI of 8.794. The predicted proteins (**Figure 1**) have conserved MADS-MEF2-like and K-box domains, which are typical characteristics of MIKC-type MADS-box proteins, there are ten amino acids different between these two MADS-box proteins

### Chromosomal Localization of *TaMADS1* and *TaMADS2*

To assign the two MADS-box genes to the wheat genome, we used Chinese Spring nulli-tetrasomic lines for chromosomal localization. Specific primers for *TaMADS1* and *TaMADS2* were designed (Supplementary Table S1) and PCR was performed using DNA from the nulli-tetrasomic lines as templates. The results (Supplementary Figure S1) showed that N3A/T3B had no *TaMADS1* band and the N3B/T3D lane had no *TaMADS2* band, indicating that *TaMADS1* and *TaMADS2* were localized on wheat chromosomes 3A and 3B, respectively, thus these were designated as *TaAG-A* and *TaAG-B*.

### Phylogenetic Analysis of *TaAG-A* and *TaAG-B*

**FIGURE 2:**
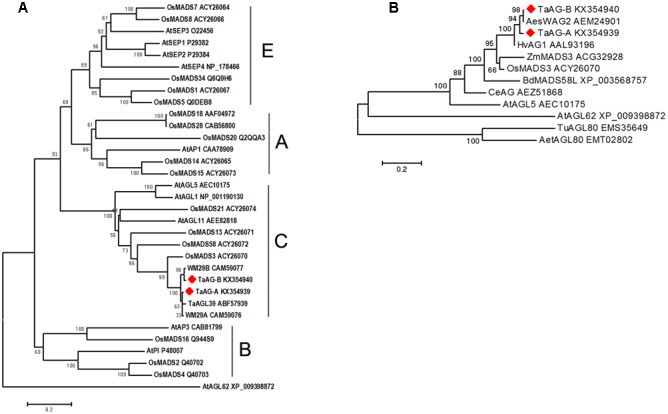
**(A)** Evolutionary relationships of MADS-box amino acid sequences in rice, *Arabidopsis*, and wheat. **(B)** Phylogenetic analysis of MADS-box proteins in different plants. Evolutionary analyses were conducted using MEGA 6 software with the neighbor-joining method [bootstrap test (1000 replicates) and Poisson correction method were used]. The phylogenetic tree was constructed using the MADS-box amino acid sequences from barley (Hv), maize (Zm), rice (Os), *B. distachyon* (Bd), *Arabidopsis thaliana* (At), *Triticum urartu* (Tu), *Aegilops speltoides* (Aes), *Aegilops tauschii* (Aet), and *Cymbidium eburneum* (Ce).

We performed phylogenetic analysis using amino acid sequences derived from rice, *Arabidopsis*, and wheat MADS-box genes. The results (**Figure 2A**) showed that TaAG-A and TaAG-B belong to the AG subfamily C group of MADS-box family proteins and have very high identity to MIKC-type MADS-box transcription factors WM29B (CAM59077) and TaAGL39 (ABF57939) from wheat and 93% identity with OsMADS3 (ACY26070) from rice.

Further phylogenetic analysis of AG subfamily proteins (**Figure 2B**) from wheat, barley, rice, maize, *Brachypodium distachyon*, *T. urartu*, and *Aegilops tauschii* showed that *TaAG-A* and *TaAG-B* are closely related to AesWAG-2 (AEM24901) from *Aegilops tauschii* and HvAG1 (AAL93196) from barley.

### Expression Analysis of *TaAG-A* and *TaAG-B*

**FIGURE 3:**
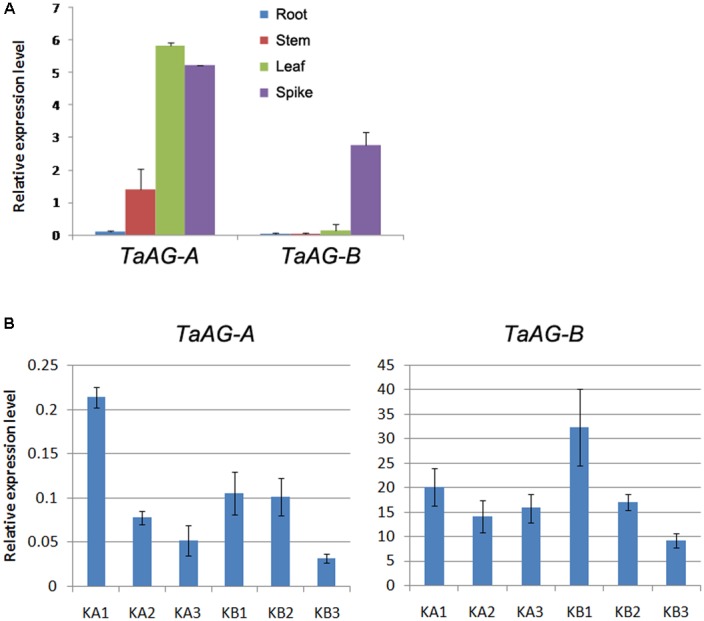
**(A)** Relative expression levels of *TaAG-A* and *TaAG-B* in tissues of Chinese Spring wheat. **(B)** Relative expression levels of *TaAG-A* and *TaAG-B* in wheat anthers at various developmental stages. KA1–3, mononucleate, dinucleate, and trinucleate stages of the wheat K-type CMS line, respectively. KB1–3, mononucleate, dinucleate, and trinucleate stages of the maintainer line, respectively.

To assess the expression patterns of *TaAG-A* and *TaAG-B* in wheat, we performed qRT-PCR analysis using RNA extracted from roots, stems, leaves, and young spikes of Chinese Spring wheat. Anthers were collected from the wheat K-type CMS line at the uni-, di-, and tri-nucleate stages and the maintainer line. The results (**Figure 3A**) demonstrate that *TaAG-A* and *TaAG-B* had similar expression patterns in different wheat tissues, but their expression levels differed in different tissues. *TaAG-A* had very high expression levels in young spikes and leaves and very low expression levels in roots, while *TaAG-B* was most abundant in young spikes with very low expression in roots, stems, and leaves.

The expression levels of *TaAG-A* and *TaAG-B* also showed similar trends at different developmental stages in anthers of the same wheat variety (**Figure 3B**). Both genes could be detected at all developmental stages in anthers, but *TaAG-B* transcripts were more abundant than those of *TaAG-A.* From the di-nucleate to tri-nucleate stages, the expression levels of *TaAG-B* were upregulated in the CMS line, but downregulated in the maintainer line. Additionally, the expression levels of *TaAG-A* and *TaAG-B* in the CMS line at the trinucleate stage were higher than in the maintainer line. These results demonstrate that *TaAG-A* and *TaAG-B* have different expression patterns between the CMS line and maintainer line at different anther developmental stages, especially at the trinucleate stage. Higher transcription of *TaAG-A* and *TaAG-B* may be closely related to male sterility in the CMS line.

### Functional Analysis of *TaAG-A* and *TaAG-B*

**FIGURE 4:**
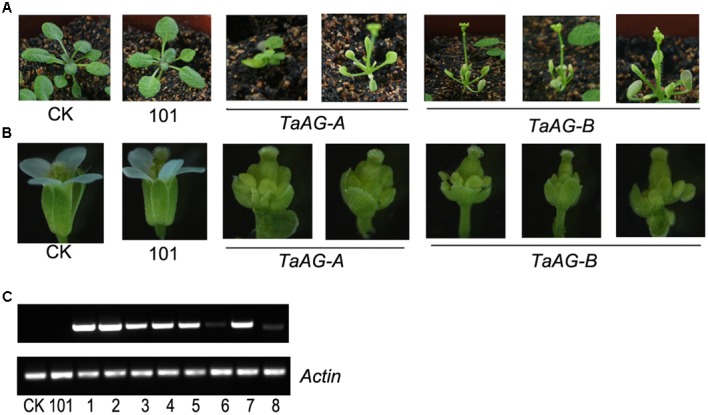
Phenotypes and expression analysis of TaAG-A and TaAG-B transgenic *Arabidopsis.* **(A)** Phenotypes of transgenic plants 16 days after transplantation. **(B)** Phenotypes of buds. **(C)** Expression analysis. CK, *Arabidopsis* wild-type Col-0 plants; 101, pEarleyGate 101 transgenic *Arabidopsis* plants; lanes 1–5, pEarleyGate 101-*TaAG-B* transgenic *Arabidopsis* plants (phenotype changed); lane 6, pEarleyGate 101-*TaAG-B* transgenic *Arabidopsis* plants (phenotype unchanged); lanes 7–8, pEarleyGate 101-*TaAG-A* transgenic *Arabidopsis* plants (phenotype changed and phenotype unchanged, respectively).

To further investigate the functions of the *TaAG-A* and *TaAG-B* genes, we constructed overexpression plasmids (pEarleyGate 101-*TaAG-A* and pEarleyGate 101-*TaAG-B*) using the Gateway-compatible vector cloning system. Plasmid constructs were individually transformed into *Arabidopsis* wild-type Col-0. More than 20 transformants were obtained and T3 homozygous plants were used for phenotypic analysis (**Figure 4A**). We compared the vegetative growth of Col-0 and the pEarleyGate 101 transformants. Both the pEarleyGate 101-*TaAG-A* and pEarleyGate 101-*TaAG-B* transgenic plants were emaciated, dwarfed, and abnormal with inwardly curled young leaves, tiny yellow leaves, and advanced reproductive growth. At the ripening stage, pEarleyGate 101-*TaAG-A* and pEarleyGate 101-*TaAG-B* plants displayed abnormal growth and development, and exhibited delayed flowering and podding.

We observed the buds of transgenic *Arabidopsis* under a stereomicroscope (**Figure 4B**) in comparison with Col-0 buds. The pEarleyGate 101-*TaAG-B* plants had short and abnormal sepals, petals, and stamens; expanded pistils; and no selfing fructification.

We performed semi-quantitative RT-PCR analysis of flowers from transgenic plants compared with normal plants using *Actin* expression as a control (**Figure 4C**). *TaAG-A* and *TaAG-B* transcript levels were higher in plants with abnormal phenotypes, indicating that *TaAG-A* and *TaAG-B* expression had a great influence on pistil and stamen development of transgenic *Arabidopsis*. In addition, *TaAG-B* induced abnormal development more readily than *TaAG-A* in transgenic *Arabidopsis*.

### Evidence for the Involvement of *TaAG-A* and *TaAG-B* in Wheat Male Sterility

**FIGURE 5:**
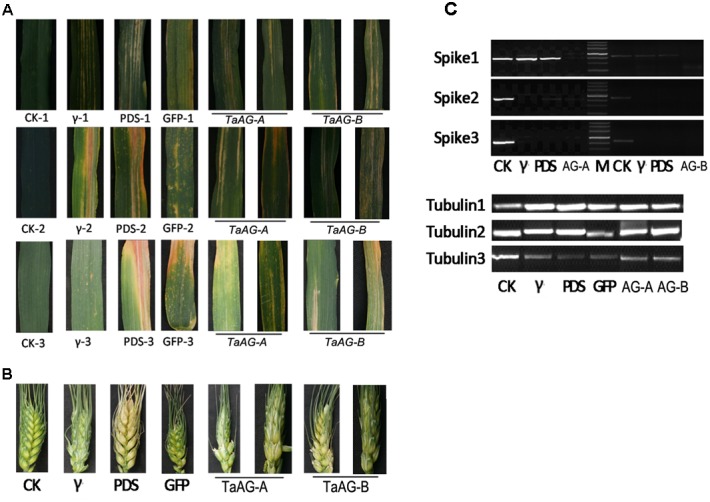
Phenotypes of virus-infected wheat leaves and spikes. **(A)** Leaf samples. **(B)** Spike samples. **(C)** Expression analysis of *TaAG-A* and *TaAG-B* genes in fertile wheat lines. CK, uninfected wheat plants; γ, wheat plants infected by RNAγb; PDS, wheat plants infected by RNAγb:TaPDS; GFP, wheat plants infected by RNAγb:GFP; 1, 2, and 3 represent different sampling stages.

To obtain more direct evidence of the functions of *TaAG-A* and *TaAG-B*, we performed virus-induced gene silencing (VIGS) developed with the barley stripe mosaic virus (BSMV) to decrease their transcript levels. Uninfected wheat plants, wheat plants infected by RNA_γb_, RNA_γb:TaPDS_, and RNA_γb:GFP_ served as controls in the inoculation assays. The fourth leaf (**Figure 5A**) and spikes (**Figure 5B**) of inoculated plants were observed at 12, 16, and 20 days after inoculation. The leaves of the wheat plants infected by RNAγb had striped spots, while wheat plants inoculated with BSMV:TaPDS had white leaves, and the leaves of BSMV:GFP-inoculated plants produced yellow, diseased spots, indicating that the BSMV experiments were successful. In plants inoculated with BSMV:TaAG-A and BSMV:TaAG-B, leaf surfaces had striped diseased spots, which were more obvious at 16 and 20 days after inoculation.

The VIGS assays also provided a visual indication of the occurrence of *TaAG-A* and *TaAG-B* gene silencing in spikes after virus inoculation. The spikes of plants inoculated with BSMV:*TaAG-A* and BSMV:*TaAG-B* were wizened and the ripening rate was notably reduced.

To further verify the involvement of *TaAG-A* and *TaAG-B* in wheat male sterility, we assessed the relative transcript levels of the *TaAG-A* and *TaAG-B* genes in spikes by semi-quantitative RT-PCR analysis with *tubulin* as a control (**Figure 5C**). *TaAG-A* and *TaAG-B* were silenced in spikes after viral inoculation. At 16 and 20 days after inoculation, the relative transcript levels in spikes inoculated with RNA_γb_ and RNA_γb:GFP_ were decreased compared to the control, indicating that BSMV infection affected the growth and development of plants.

## Discussion

Cytoplasmic male sterility is a maternally inherited trait resulting in failure to produce functional pollen. It is generally thought that cytoplasmic male sterility is associated with the rearrangement of mitochondrial genomes (Schnable and Wise, 1998; Hanson and Bentolila, 2004). Recently, some MADS-box genes related to floral organ development were reported in wheat to be targets for floral organ homeotic transformation regulated by mitochondrial retrograde regulation, which is the communication pathway from mitochondria to the nucleus (Meguro et al., 2003; Hama et al., 2004). We found from our previous microarray results that the expression level of an MIKC-type MADS-box transcription factor, Ta.4147, in the CMS line was higher than in the maintainer line. To elucidate the relationship between the MADS-box gene and male sterility of the wheat K-type CMS line in this study, we isolated two MADS-box genes named *TaAG-A* and *TaAG-B* from uninucleate pollen and assigned them to chromosomes 3A and 3B, respectively. Their predicted amino acid sequences had typical conserved domains of MADS-MEF2-like and K-box of MADS-box proteins. Phylogenetic analysis showed that *TaAG-A* and *TaAG-B* belong to the AG subfamily C group of the MADS-box family. The hexaploid wheat (AABBDD) genome contains triplicate homoeologs of each gene derived from the ancestral diploid *T. urartu* (AA), *Aegilops speltoides* (BB), and *Aegilops tauschii* (DD). However, we only isolated two homoeologs, *TaAG-A* and *TaAG-B*, using RT-PCR in this study, indicating that transcription of the D genome homoeolog did not occur. Tanaka et al. (2015) also reported that the homoeologs of *WAG1* (*wheat AGAMOUS1*) on the A and B genomes were expressed, while expression of its D genome homoeolog was suppressed.

According to the ABC(DE) and ‘floral quartets’ models, the combination BCE specifies the formation of stamens and the combination CE determines the development of carpels (Theißen, 2001). In *Arabidopsis*, *AG* is required to specify the identity of stamens and pistils (Yanofsky et al., 1990). Mutation in a class-C gene leads to homeotic transformations of the organs of region C (i.e., stamens and carpels) into perianth organs (i.e., petals and sepals, respectively). The wheat inflorescence, which is composed of spikelets, is quite different from the inflorescences of dicots. Stamens are transformed into pistil-like structures, which in turn contain ovule-like structures in the alloplasmic wheat line (cr)-CSdt7BS (Murai et al., 2002). Meguro et al. (2003) subsequently reported that an *AG* homolog, *WAG*, is associated with pistillody caused by nuclear-cytoplasm interaction in the alloplasmic lines of common wheat with the *Aegilops crassa* cytoplasm. The *WAG* gene reported by Meguro et al. (2003) is known as *WAG1*/*TaAG-1* and *WAG2* is known as *TaAG-2*/*TaAGL39* (Zhao et al., 2006; Paolacci et al., 2007). Phylogenetic analysis showed that *WAG1* and *WAG2* are orthologs of rice *OsMADS58* and *OsMADS3*, respectively. *OsMADS3*, *ZMM2*, *WM29A*, *TaAGL39*, *WM29B*, and *HvAG1* were clustered in the *WAG2* clade (Murai, 2013). In this study, *TaAG-A* and *TaAG-B* were closely related to *WM29A*, *WM29B*, *TaAGL39*, and *OsMADS3*, indicating that *TaAG-A* and *TaAG-B* may play similar roles to *WAG2*, *OsMADS3*, and *ZMM2*. *OsMADS3* plays a predominant role in inhibiting lodicule development and in specifying stamen identity (Kang et al., 1995). *ZMM2* participates in regulating the formation of stamens and carpels (Mena et al., 1996). Mizumoto et al. (2009) found that *WAG-2* is preferentially expressed in the central region of pistils rather than developing stamens during floral organ development. Thus, the function of *WAG-2* is also associated with pistillody. Based on these studies, we deduced that *TaAG-A* and *TaAG-B* may be involved in the development of pistil-like stamens in the K-type CMS line.

To verify our hypothesis, we performed qRT-PCR analysis to assess the expression patterns of *TaAG-A* and *TaAG-B* in wheat. In Chinese Spring wheat at the heading stage, *TaAG-A* had relatively high expression levels in leaves and spikes, and low levels in roots. Similarly, *TaAG-B* had relatively high expression levels in spikes and low levels in roots, stems, and leaves. Our results suggest that the product of the *TaAG-A* transcript is involved in leaf and spike development, while the product of *TaAG-B* functions mainly in spike development. The expression patterns of *WAG1* homoeologs, with high expression of *WAG1-A*, very low expression of *WAG1-B*, and silencing of *WAG1-D* in young spikes, are consistent and reproducible features of hexaploid lines (Tanaka et al., 2015). However, *AG* orthologous genes, such as *AG* (*Arabidopsis*, Yanofsky et al., 1990), *ZAG1* (maize, Schmidt et al., 1993), and *OsMADS3* (rice, Kang et al., 1995), were not expressed in any vegetative tissues. The expression of TaAGL39 was detectable in 16-h, 20-h, and 28-h embryos of imbibed seeds, stems at the jointing stage, and developing seeds at 6 and 12 days after pollination (Zhao et al., 2006). In anthers at different stages (mononucleate, dinucleate, and trinucleate) collected from the K-type CMS line and the maintainer line, *TaAG-B* expression increased from the dinucleate to the trinucleate pollen stages in the CMS line, both *TaAG-A* and *TaAG-B* downregulated in the maintainer line. Meguro et al. (2003) also found that *WAG* was preferentially expressed during late stages in developing spikes.

To further investigate the function of *TaAG-A* and *TaAG-B* genes, we overexpressed *TaAG-A* and *TaAG-B* in *Arabidopsis* and silenced *TaAG-A* and *TaAG-B* in fertile wheat lines using BSMV-VIGS. The transgenic *Arabidopsis* showed earlier reproductive development, premature mortality, and abnormal buds, stamens, and stigmas. In addition, the higher the *TaAG-A* and *TaAG-B* expression levels were, the greater the deformity. Expression analysis of transgenic *Arabidopsis* indicated that *TaAG-A* and *TaAG-B* are involved in pistil and stamen development. BSMV-VIGS is an effective reverse genetics tool to investigate the genetic function of important genes in wheat (Scofield et al., 2005; Zhou et al., 2007). The *TaAG-A* and *TaAG-B* silenced plants had green and yellow striped leaves, emaciated spikes, and decreased selfing seed set rates. These results suggest that *TaAG-A* and *TaAG-B* may play roles in male sterility in the wheat CMS line.

## Author Contributions

WY and AZ designed the study and provide guidance on the whole study. XL, JL, and MP carried out the plant materials culture, qRT-PCR analysis, data analysis, and drafted the manuscript. DL, JS, KZ, and LH prepared plant materials. DL and AM provided the value comments and revised the grammar of the manuscript. All authors read and approved the final manuscript.

## Conflict of Interest Statement

The authors declare that the research was conducted in the absence of any commercial or financial relationships that could be construed as a potential conflict of interest.

## Footnotes

1 http://plants.ensembl.org/Triticum_urartu/Tools/Blast?db=core

2 http://www.ncbi.nlm.nih.gov/Structure/cdd/wrpsb.cgi

## References

[B1] Ai Y. Zhang Q. Wang W. Zhang C. Cao Z. Bao M. (2016). Transcriptomic analysis of differentially expressed genes during flower organ development in genetic male sterile and male fertile *Tagetes erecta* by digital gene-expression profiling. *PLoS ONE* 11:e0150892 10.1371/journal.pone.0150892 PMC4777371 26939127

[B2] Clough S. J. Bent A. F. (1998). Floral dip: a simplified method for *Agrobacterium*- mediated transformation of *Arabidopsis thaliana*. *Plant J.* 16 735–743. 10.1046/j.1365-313x.1998.00343.x 10069079

[B3] Coen E. S. Meyerowitz E. M. (1991). The war of the whorls: genetic interactions controlling flower development. *Nature* 353 31–37. 10.1038/353031a0 1715520

[B4] De Bodt S. Raes J. Van de Peer Y. Theißen G. (2003). And then there were many: MADS goes genomic. *Trends Plant Sci.* 8 475–483. 10.1016/j.tplants.2003.09.006 14557044

[B5] Duan K. Li L. Hu P. Xu S. P. Xu Z. H. Xue H. W. (2006). A brassinolide-suppressed rice MADS-box transcription factor, OsMDP1, has a negative regulatory role in BR signaling. *Plant J.* 47 519–531. 10.1111/j.1365-313X.2006.02804.x 16827922

[B6] Duvick D. N. (1959). The use of cytoplasmic male-sterility in hybrid seed production. *Econ. Bot.* 13 167–195. 10.1007/BF02860581

[B7] Goto K. Meyerowitz E. M. (1994). Function and regulation of the *Arabidopsis* floral homeotic gene *PISTILLATA*. *Genes Dev.* 8 1548–1560. 10.1101/gad.8.13.1548 7958839

[B8] Hama E. Takumi S. Ogihara Y. Murai K. (2004). Pistillody is caused by alterations to the class-B MADS-box gene expression pattern in alloplasmic wheats. *Planta* 218 712–720. 10.1007/s00425-003-1157-6 14652757

[B9] Hanson M. R. Bentolila S. (2004). Interactions of mitochondrial and nuclear genes that affect male gametophyte development. *Plant Cell* 16(Suppl.), S154–S169. 10.1105/tpc.015966 15131248 PMC2643387

[B10] Hirabayashi C. Murai K. (2009). Class C MADS-box gene *AGAMOUS* was duplicated in the wheat genome. *Wheat Inf. Serv.* 107 13–16.

[B11] Huang Y. Yang W. Pei Z. Guo X. Liu D. Sun J. (2012). The genes for gibberellin biosynthesis in wheat. *Funct. Integr. Genomics* 12 199–206. 10.1007/s10142-011-0243-2 21853379

[B12] Jack T. Brockman L. L. Meyerowitz E. M. (1992). The homeotic gene *APETALA3* of *Arabidopsis thaliana* encodes a MADS box and is expressed in petals and stamens. *Cell* 68 683–697. 10.1016/0092-8674(92)90144-2 1346756

[B13] Jofuku K. D. den Boer B. G. Van Montagu M. Okamuro J. K. (1994). Control of *Arabidopsis* flower and seed development by the homeotic gene *APETALA2*. *Plant Cell* 6 1211–1225. 10.1105/tpc.6.9.1211 7919989 PMC160514

[B14] Kang H. G. Noh Y. S. Chung Y. Y. Costa M. A. An K. An G. (1995). Phenotypic alterations of petal and sepal by ectopic expression of a rice MADS box gene in tobacco. *Plant Mol. Biol.* 29 1–10. 10.1007/BF00019114 7579155

[B15] Li A. Yang W. Li S. Liu D. Guo X. Sun J. (2013). Molecular characterization of three *GIBBERELLIN-INSENSITIVE DWARF1* homologous genes in hexaploid wheat. *J. Plant Physiol.* 170 432–443. 10.1016/j.jplph.2012.11.010 23261263

[B16] Mandel M. A. Gustafson-Brown C. Savidge B. Yanofsky M. F. (1992). Molecular characterization of the *Arabidopsis* floral homeotic gene *APETALA1*. *Nature* 360 273–277. 10.1038/360273a0 1359429

[B17] Meguro A. Takumi S. Ogihara Y. Murai K. (2003). *WAG*, a wheat *AGAMOUS* homolog, is associated with development of pistil-like stamens in alloplasmic wheats. *Sex. Plant Reprod.* 15 221–230.

[B18] Mena M. Ambrose B. A. Meeley R. B. Briggs S. P. Yanofsky M. F. Schmidt R. J. (1996). Diversification of C-function activity in maize flower development. *Science* 274 1537–1540. 10.1126/science.274.5292.1537 8929416

[B19] Mizumoto K. Hatano H. Hirabayashi C. Murai K. Takumi S. (2009). Altered expression of wheat *AINTEGUMENTA* homolog, *WANT-1*, in pistil and pistil-like transformed stamen of an alloplasmic line with *Aegilops crassa* cytoplasm. *Dev. Genes Evol.* 219 175–187. 10.1007/s00427-009-0275-y 19255779

[B20] Münster T. Pahnke J. Di Rosa A. Kim J. T. Martin W. Saedler H. (1997). Floral homeotic genes were recruited from homologous MADS-box genes preexisting in the common ancestor of ferns and seed plants. *Proc. Natl. Acad. Sci. U.S.A.* 94 2415–2420. 10.1073/pnas.94.6.2415 9122209 PMC20102

[B21] Murai K. (2013). Homeotic genes and the ABCDE model for floral organ formation in wheat. *Plants* 2 379–395. 10.3390/plants2030379 27137382 PMC4844379

[B22] Murai K. Miyama M. E. Kato H. Takumi S. Ogihara Y. (2003). *WAP1*, a wheat *APETALA1* homolog, plays a central role in the phase transition from vegetative to reproductive growth. *Plant Cell Physiol.* 44 1255–1265. 10.1093/pcp/pcg171 14701921

[B23] Murai K. Takumi S. Koga H. Ogihara Y. (2002). Pistillody, homeotic transformation of stamens into pistil-like structures, caused by nuclear-cytoplasm interaction in wheat. *Plant J.* 29 169–181. 10.1046/j.0960-7412.2001.01203.x 11851918

[B24] Murashige T. Skoog F. (1962). A revised medium for rapid growth and bio-assays with tobacco tissue cultures. *Physiol. Plant.* 15 473–497. 10.1111/j.1399-3054.1962.tb08052.x

[B25] Ng M. Yanofsky M. F. (2001). Function and evolution of the plant MADS-box gene family. *Nat. Rev. Genet.* 2 186–195. 10.1038/35056041 11256070

[B26] Paolacci A. R. Tanzarella O. A. Porceddu E. Ciaffi M. (2009). Identification and validation of reference genes for quantitative RT-PCR normalization in wheat. *BMC Mol. Biol.* 10:11 10.1186/1471-2199-10-11 PMC2667184 19232096

[B27] Paolacci A. R. Tanzarella O. A. Porceddu E. Varotto S. Ciaffi M. (2007). Molecular and phylogenetic analysis of MADS-box genes of MIKC type and chromosome location of *SEP*-like gene in wheat (*Triticum aestivum* L.). *Mol. Genet. Genomics* 278 689–708. 10.1007/s00438-007-0285-2 17846794

[B28] Saghai-Maroof M. A. Soliman K. M. Jorgensen R. A. Allard R. W. (1984). Ribosomal DNA spacer-length polymorphism in barley: mendelian inheritance, chromosomal location, and population dynamics. *Proc. Natl. Acad. Sci. U.S.A.* 81 8014–8018. 10.1073/pnas.81.24.8014 6096873 PMC392284

[B29] Schmidt R. J. Veit B. Mandel M. A. Mena M. Hake S. Yanofsky M. F. (1993). Identification and molecular characterization of *ZAG1*, the maize homolog of the *Arabidopsis* floral homeotic gene *AGAMOUS*. *Plant Cell* 5 729–737. 10.1105/tpc.5.7.729 8103379 PMC160311

[B30] Schnable P. S. Wise R. P. (1998). The molecular basis of cytoplasmic male sterility and fertility restoration. *Trends Plant Sci.* 3 175–180. 10.1016/S1360-1385(98)01235-7

[B31] Schwarz-Sommer Z. Huijser P. Nacken W. Saedler H. Sommer H. (1990). Genetic control of flower development by homeotic genes in *Antirrhinum majus*. *Science* 250 931–936. 10.1126/science.250.4983.931 17746916

[B32] Scofield S. R. Huang L. Brandt A. S. Gill B. S. (2005). Development of a virus-induced gene-silencing system for hexaploid wheat and its use in functional analysis of the *Lr21*-mediated leaf rust resistance pathway. *Plant Physiol.* 138 2165–2173. 10.1104/pp.105.061861 16024691 PMC1183404

[B33] Tanaka M. Tanaka H. Shitsukawa N. Kitagawa S. Takumi S. Murai K. (2015). Homoeologous copy-specific expression patterns of MADS-box genes for floral formation in allopolyploid wheat. *Genes Genet. Syst.* 90 217–229. 10.1266/ggs.15-00029 26616759

[B34] Theißen G. (2001). Development of floral organ identity: stories from the MADS house. *Curr. Opin. Plant Biol.* 4 75–85. 10.1016/S1369-5266(00)00139-4 11163172

[B35] Tsunewaki K. Mukai Y. Endo E. R. (1978). “On the decent of the cytoplasm of polyploid species in *Triticum* and *Aegilops*,” in *Proceedings of the 5th International Wheat Genetics Symposium, New Delhi*, 261–272.

[B36] Wang Q. H. Yang Z. J. Wei S. H. Jiang Z. Y. Yang Y. F. Hu Z. S. (2015). Molecular cloning, characterization and expression analysis of WAG-1 in the pistillody line of common wheat. *Genet. Mol. Res.* 14 12455–12465. 10.4238/2015.October.16.12 26505395

[B37] Wei S. Peng Z. Zhou Y. Yang Z. Wu K. Ouyang Z. (2011). Nucleotide diversity and molecular evolution of the *WAG-2* gene in common wheat (*Triticum aestivum* L) and its relatives. *Genet. Mol. Biol.* 34 606–615. 10.1590/S1415-47572011000400013 22215965 PMC3229116

[B38] Weigel D. Meyerowitz E. M. (1994). The ABCs of floral homeotic genes. *Cell* 78 203–209. 10.1016/0092-8674(94)90291-7 7913881

[B39] Yanofsky M. F. Ma H. Bowman J. L. Drews G. N. Feldman K. A. Meyerowitz E. M. (1990). The protein encoded by the *Arabidopsis* homeotic gene *agamous* resembles transcription factors. *Nature* 346 35–39. 10.1038/346035a0 1973265

[B40] Zhao T. Ni Z. Dai Y. Yao Y. Nie X. Sun Q. (2006). Characterization and expression of 42 MADS-box genes in wheat (*Triticum aestivum* L.). *Mol. Genet. Genomics* 276 334–350. 10.1007/s00438-006-0147-3 16858583

[B41] Zhao Y. X. Cheng Z. J. Zhang X. S. (2005). Overexpression of *TaMADS1*, a *SEPALLATA*-like gene in wheat, causes early flowering and the abnormal development of floral organs in *Arabidopsis*. *Planta* 223 698–707. 10.1007/s00425-005-0123-x 16177912

[B42] Zhou H. Li S. Deng Z. Wang X. Chen T. Zhang J. (2007). Molecular analysis of three new receptor-like kinase genes from hexaploid wheat and evidence for their participation in the wheat hypersensitive response to stripe rust fungus infection. *Plant J.* 52 420–434. 10.1111/j.1365-313X.2007.03246.x 17764502

