# Supplementary material for: Cloning and Functional Analysis of MADS-box Genes, TaAG-A and TaAG-B, from a Wheat K-type Cytoplasmic Male Sterile Line
Source: Front Plant Sci. 2017 Jun 20;8:1081. doi: 10.3389/fpls.2017.01081 (PMC5476771; doi:10.3389/fpls.2017.01081)
Supplement: Supplementary file 1 [file Table_1.DOCX]

**Supplemental information**

**Table S1. Primers used in this study**

| Name | Sequence (5’ to 3’) | Purpose |
| --- | --- | --- |
| TaMADS1.CF | ATGATGAGCATGATGGCCGAT | Cloning for MADS-box genes |
| TaMADS1.CR | CTAGTTGAAGTACTGCTGGCCGA |  |
| TaMADS2.CF | CTAGCTCACAGAGGCACGTT |  |
| TaMADS2.CR | ACAGCACGGCGACAGTATTA |  |
| TaMADS1.LEF | ATGATGGCGTCAGGGTCG | Chromosomal localization and expression analysis for *TaAG-A* and *TaAG-B* genes |
| TaMADS1.LER | GACATTCCAGGCACACTGC |  |
| TaMADS2.LEF | AGCAGCAGCATTACTCCCAAC |  |
| TaMADS2.LER | TTTACCACCGTAGAACGAGCAAC |  |
| Ta4045-EF | CCTGCCCCGTACAACCTTGAG |  |
| Ta4045-ER | CACCGTTGCGATAGTCCTGAAAC |  |
| TaAG-AB.101F | GGGGACAAGTTTGTACAAAAAAGCAGGCTTC ATGATGAGCATGATGGCCGAT | Construct for pEarleyGate 101-*TaAG-A* and pEarleyGate 101-*TaAG-B* |
| TaAG-AB.101R | GGGGACCACTTTGTACAAGAAAGCTGGGTC GTTGAAGTACTGCTGGCCGAGCT |  |
| 101.F | CGCACAATCCCACTATCC | Detect for *Arabidopsis* transformants |
| 101.R | GTTTACGTCGCCGTCCAG |  |
| TaAG-AB.SF | GCAGAAGTCAATGCCCAGTA | Semi-quantitative RT-PCR for *Arabidopsis* transformants |
| TaAG-AB.SR | TCAGACACCTTGCTCCTCA |  |
| Actin.F | TCTCCCGCTATGTATGTCGCC |  |
| Actin.R | GTCACGTCCAGCAAGGTCAAGA |  |
| TaAG-A-*Nhe*I-F | CTAGCTAGCATAACATTTACCTGAGGAGCAA | Construct for RNA_γb:TaAG-Aas_and RNA_γb:TaAG-Bas_ |
| TaAG-A-*Nhe*I-R | CTAGCTAGCGAGCTGAAGGGCAGTTGG |  |
| TaAG-B-*Nhe*I-F | CTAGCTAGCTAACATTTACCTGAGGAGCAAG |  |
| TaAG-B-*Nhe*I-R | CTAGCTAGCGCACGGCGACAGTATTATCTAG |  |

F, forward primer; R, reverse primer; the underlined regions were the sites for *attB* adapter or restriction digest.

**Table S2. Two TaMADS-box genes isolated in this study**

| Characters | *TaMADS1* | *TaMADS2* |
| --- | --- | --- |
| CDS(bp) | 819 | 831 |
| Protein (amino acid) | 272 | 276 |
| Molecular weight (kDa) | 30.8 | 31.4 |
| pI | 9.072 | 8.794 |

Fig S1 **Chromosomal localization of *TaAG-A* and *TaAG-B.***

Lanes 1–23, represent the 21 Chinese Spring nullisomic-tetrasomic lines (N1A/T1D, N1B/T1D, N1D/T1A, N2A/T2D, N2B/T2D, N2D/T2A, N3A/T3B, N3B/T3D, N3D/T3A, N4A/T4B, N4B/T4D, N4D/T4A, N5A/T5D, N5B/T5D, N5D/T5B, N6A/T6B, N6B/T6D, N6D/T6B, N7A/T7D, N7B/T7D, N7D/T7B), Chinese Spring, and the negative control, respectively.
