# Supplementary figures and images for: Cloning and Functional Analysis of MADS-box Genes, TaAG-A and TaAG-B, from a Wheat K-type Cytoplasmic Male Sterile Line
Source: Front Plant Sci. 2017 Jun 20;8:1081. doi: 10.3389/fpls.2017.01081 (PMC5476771; doi:10.3389/fpls.2017.01081)

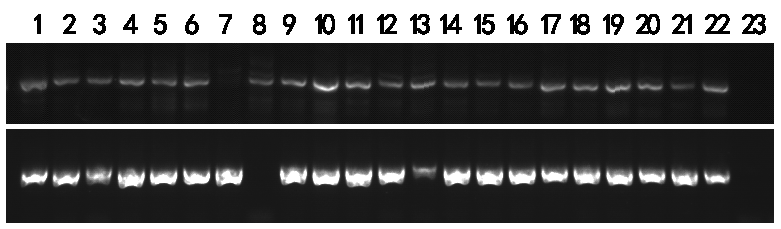

Supplement: Supplementary file 2 [file Image_1.TIF]
